# Brief Report: Emotion Regulation Influences on Internalizing and Externalizing Symptoms Across the Normative-Clinical Continuum

**DOI:** 10.3389/fpsyt.2021.693570

**Published:** 2021-07-21

**Authors:** Ru Ying Cai, Antonio Y. Hardan, Jennifer M. Phillips, Thomas W. Frazier, Mirko Uljarević

**Affiliations:** ^1^Aspect Research Centre for Autism Practice, Autism Spectrum Australia, Melbourne, VIC, Australia; ^2^Faculty of Medicine, Nursing and Health Sciences, School of Psychological Sciences, Monash University, Melbourne, VIC, Australia; ^3^Department of Psychiatry and Behavioral Sciences, Child and Adolescent Psychiatry, School of Medicine, Stanford University, Stanford, CA, United States; ^4^Department of Psychology, John Carroll University, Cleveland, OH, United States; ^5^Autism Speaks, Independence, OH, United States; ^6^Faculty of Medicine, Dentistry, and Health Sciences, Melbourne School of Psychological Sciences, University of Melbourne, Melbourne, VIC, Australia; ^7^School of Psychology and Public Health, La Trobe University, Melbourne, VIC, Australia

**Keywords:** emotion regulation, transdiagnostic, internalizing, externalizing, adolescent, autism, ADHD, anxiety

## Abstract

Emotion regulation is theorized to be a transdiagnostic process and has been empirically shown to be associated with various mental health and neurodevelopmental conditions. However, the relationship between emotion regulation and internalizing and externalizing symptoms has yet to be characterized in a sample of individuals spanning normative and atypical development. Therefore, this study aimed to provide initial evidence for emotion regulation as a transdiagnostic process of internalizing and externalizing symptoms in a community sample of adolescents with and without neuropsychiatric and neurodevelopmental conditions. The sample consisted of 1,705 caregivers of adolescents aged between 11 and 17 years (*M*_*age*_ = 14.53, *SD*_*age*_ = 1.96). Adolescents were typically developing or had a caregiver-reported diagnosis of autism spectrum disorder, attention-deficit hyperactivity disorder, or anxiety. The typically developing adolescents had significantly better caregiver-reported emotion regulation than adolescents with caregiver-reported neuropsychiatric and neurodevelopmental conditions. Additionally, emotion dysregulation significantly and positively correlated with and predicted internalizing and externalizing symptoms within each subgroup. Importantly, emotion dysregulation had a unique contribution to individual differences in the severity of internalizing and externalizing symptoms, above and beyond the diagnostic status. The research and translational implications of the study findings are discussed.

## Introduction

The dimensional frameworks such as the National Institute of Mental Health Research Domain Criteria (RDoC) (1) suggest a set of basic, neurobiologically valid dimensions of functioning that span a full range of human behaviors and represent building blocks of normative functioning and, if disrupted, can result in specific symptoms or groups of symptoms seen across a range of neuropsychiatric and neurodevelopment disorders (2). Dimensional models lend themselves to tackling one of the main limitations of the current diagnostic systems—the fact that most symptoms are not confined to specific, categorically defined mental disorders but rather occur across a range of specific conditions (3). These symptoms are also distributed throughout the general population (4, 5). Specific constructs within the cognitive and negative valence RDoC domains such as executive functioning (6, 7) and intolerance of certainty (8) have been shown to serve as general psychopathology factors. One of the critical implications of these recent studies is that focusing on specific dimensional constructs that represent risk factors for the development and maintenance of certain symptoms or groups of symptoms, irrespective of categorical diagnostic status, is effective for treatment development (2). Therefore, studying specific dimensional constructs across normative and clinical samples is a potentially fruitful approach to defining, understanding, and treating mental disorders.

Emotion regulation is a critical transdiagnostic process with well-defined biological, cognitive, and neural underpinnings (9–11). Emotion regulation is a complex process that involves the monitoring and modification of emotional responses (12). It allows individuals to modify the intensity, duration, and types of emotions experienced (13). Emotion regulation processes can be explicit (deliberate) or implicit (automatic) (14). Automatic emotion regulation occurs when emotions are regulated without conscious awareness of one's goal to modify emotions (14, 15). People use various strategies within and across situations to regulate emotions (16, 17). The development of regulatory systems is non-linear (18) due to a maladaptive shift in emotion regulation during adolescence between the ages 12 and 15 years (19). Given the robust evidence for distinct neurobiological underpinnings and proposed links with specific symptom domains, Fernandez et al. (20) proposed incorporating emotion regulation as a sixth domain in the RDoC matrix.

Several different theoretical models of emotion regulation have been put forward [see (21) for an overview]. Temporal process models focus on the temporal unfolding of emotion and emotion regulation across various stages. Strategy-based models focus on specific emotion regulation strategies (e.g., cognitive reappraisal, emotional acceptance, and expressive suppression). Ability-based models are organized around dispositional factors that facilitate emotion regulation (e.g., emotional awareness, emotional acceptance). These dispositional abilities cut across varying situations and strategies and have been linked to psychopathology development and maintenance. For instance, people who have difficulties identifying or labeling their own or other people's emotions (alexithymia) experience higher anxiety and depression symptoms (22). One of the most widely investigated ability-based models of emotion regulation (23) described the following six abilities from an emotion regulation perspective: (1) emotional awareness; (2) emotional clarity; (3) behavioral regulation; (4) engagement in goal-directed cognition and behavior when distressed; (5) emotional acceptance; and (6) access to effective strategies for feeling better when distressed. Studies have shown that difficulties in the noted emotion regulation abilities, as measured by the Difficulties in Emotion Regulation Scale (DERS) (23), are related to higher severity of symptoms that characterize a wide range of mental health conditions, including generalized anxiety disorder and depression (24, 25).

In addition to being associated with neuropsychiatric conditions, impaired emotion regulation is a prominent feature of neurodevelopmental conditions such as autism spectrum disorder (ASD) and attention-deficit hyperactivity disorder (ADHD). Overall, autistic individuals tend to employ simpler and maladaptive strategies and have poorer emotion regulation abilities than non-autistic individuals (26, 27). Different aspects of the core ASD phenotype are associated with emotion dysregulation (28), with restricted and repetitive behaviors, interests and activities playing a more prominent role than socio-communicative impairments (29). People with ADHD tend to experience a range of emotion processing impairments such as failure to inhibit emotions (emotional impulsivity) and emotion dysregulation, leading some researchers to suggest that these impairments should be considered integral features of ADHD (30, 31). Emotion regulation difficulties in people with ASD and ADHD have also been shown to predict psychopathology. For instance, greater use of adaptive emotion regulation strategies has been shown to predict reduced symptoms of anxiety and depression in autistic youth and adults (32, 33). For youth diagnosed with ADHD, emotion regulation mediates the relationship between ADHD and depressive symptoms (34).

Demonstrating that emotion regulation predicts variability in internalizing and externalizing symptoms in a transdiagnostic fashion would support the notion that treatment approaches targeting emotion regulation can help reduce internalizing and externalizing symptoms irrespective of the specific categorical diagnosis. Meta-analytic reviews have provided consistent and solid evidence for the association between emotion regulation and psychopathology (35, 36). A systematic review by Sloan et al. (37) also found that regardless of clinical diagnoses (anxiety, depression, substance use, eating pathology, or borderline personality disorder) or intervention, overall emotion dysregulation and maladaptive emotion regulation strategy use significantly reduced after psychological treatments in all but two studies. Although these reviews have summarized findings on ER across conditions, primary empirical studies that were included in noted reviews have focused only on normative samples or a specific clinical condition (with or without controls). No empirical study has examined the relationship between ER and psychopathology in a sample spanning normative and atypical development. Given that emotion regulation is hypothesized to be a transdiagnostic process, it is critical to examine emotion regulation in a sample of individuals spanning normative and atypical development.

The current study aimed to characterize the relationship between caregiver-reported emotion regulation, internalizing and externalizing symptoms in a sample of adolescents spanning normative and atypical development. We focused on adolescent population, given that this period is a peak time for the onset of mental health conditions, therefore offering a key critical opportunity for well-timed, effective treatments and supports (38). Crucially, adolescents experience a maladaptive shift in emotion regulation (19). We hypothesized that typically developing (TD) adolescents would have better emotion regulation than adolescents with neuropsychiatric and neurodevelopmental conditions. Additionally, we envisaged that emotion dysregulation would significantly and positively correlate with and predict internalizing and externalizing symptoms for the entire sample as well as within each participant group (TD and clinical groups).

## Methods

### Participants

The sample consisted of 1,705 caregivers of adolescents aged between 11 and 17 years (*M*_*age*_ = 14.53, *SD*_*age*_ = 1.96; 52% male) who were recruited using Dynata, an online recruitment platform (60% mothers, 36% fathers, 4% others: grandparent, relative, stepparent, and legal guardian). One thousand three hundred and eighty-one adolescents were typically developing (TD), and for 324 adolescents, caregivers reported at least one clinical diagnosis (*n* = 118 ADHD, *n* = 113 Anxiety, and *n* = 93 ASD). Given that co-morbidity is a common feature of current diagnostic systems, we have adopted the approach of classifying adolescents based on the diagnosis with the highest impact on functioning in the current study. For instance, if a parent reported their child as having both ASD and anxiety or both ASD and ADHD, the child was classified as having ASD. Adolescents with other conditions such as Depression, Obsessive-Compulsive Disorder, and Intellectual Disorder were excluded from this sample because the sub-sample sizes of these conditions were too small for analyses. Inclusion criteria for TD children and adolescents were that they had a *T* score of 59 or lower on the Social Responsiveness Scale (SRS-2) (39). Inclusion criteria for ASD was an SRS-2 T score of 60 or greater, and for other clinical diagnoses, inclusion criteria were that they met the Strengths and Difficulties Questionnaire (SDQ) (40) total score cut-off or the cut-off score on the corresponding subscale of the SDQ (i.e., the emotional symptoms subscale for Anxiety or the hyperactivity/inattention symptoms subscale for ADHD). See Table 1 for demographic information for each group.

**Table 1:** Demographic information of groups.

	**TD**	**Anxiety**	**ADHD**	**ASD**
*n*	1,381	113	118	93
Age *M(SD)*	14.51 (1.95)	14.82 (2.02)	14.41 (1.98)	14.67 (2.11)
**Gender**				
Male	50%	41%	65%	73%
Female	50%	59%	35%	27%
**Ethnicity**				
African American	8%	8%	12%	4%
American Indian or	2%	4%	3%	3%
Alaska Native				
Asian	13%	6%	8%	8%
Hispanic/Latino	8%	15%	9%	15%
Native Hawaiian	0%	2%	2%	3%
White	81%	81%	87%	88%
Other	1%	1%	0%	1%
**Household income**				
Less than $10,000	1%	1%	2%	7%
$10,000 to $19,9999	2%	6%	10%	5%
$20,000 to $29,999	3%	6%	6%	10%
$30,000 to $49,999	9%	13%	12%	17%
$50,000 to $74,999	17%	22%	17%	15%
$75,000 to $99,999	20%	20%	19%	23%
$100,000 to $124,999	14%	7%	14%	8%
$125,000 to $149,999	11%	8%	8%	8%
$150,000 to $199,999	12%	11%	6%	3%
Over $200,000	11%	6%	6%	4%

### Procedures and Measures

The recruitment strategy followed that of previously published research and conducted recruitment online using Dynata [formerly Survey Sampling International (SSI; Shelton, CT)], an online recruitment platform that specializes in recruiting demographically representative samples for scientific research in the United States [e.g., (41–43)] that is similar to other established and reliable commercial data recruitment platform [e.g., Prolific Academic, Amazon's Mechanical Turk (44–46)]. Only individuals who did not fail any of the attention checks (47, 48) were included in the final sample. The survey responses were anonymous.

The online survey consisted of a few basic demographic questions (child's age, child's gender, child's ethnicity, relationship of caregiver to child, and gross household income) and child's current diagnosis of mental disorders. The following measures were considered for this investigation:

*The Social Responsiveness Scale, 2nd edition (SRS-2)* *(**39**)* school-age form is a 65-item parent- or teacher-report screener of ASD. The subscales of SRS-2 are social awareness, social cognition, social communication, social motivation, and restricted interests and repetitive behavior. The SRS-2 total score is expressed in raw and *T*-score format. *T*-scores below 60 are considered to be within typical range.

*The Difficulties in Emotion Regulation Scale—Parent Report (DERS-P)* *(**49**)* consists of 28 items of the original Difficulties in Emotion Regulation Scale (23), validated in two samples of parents of adolescents with ADHD. The DERS measures abilities that are important for emotion regulation. The total scores of DERS-P ranges between 28 and 140. The Cronbach's alpha for this sample was excellent (0.95).

*The Strengths and Difficulties Questionnaire (SDQ)* *(**40**)* is a 25-item parent-report measure of emotional and behavioral problems in children, with standardized norms across age groups and genders. It provides a total score (ranging from 0 to 40) as well as scores for four empirically-based syndrome scales (emotional, conduct, hyperactivity, and peer problems) that are grouped into internalizing and externalizing problems domains used here.

### Analysis Plan

Tests of normality (Kolmogorov–Smirnov statistic) were used to determine whether the distributions were normally distributed. Chi-square test of independence, Kruskal-Wallis and Mann–Whitney *U*-tests were used to compare the groups on age, gender, emotion regulation difficulties, internalizing, and externalizing symptoms. Correlation analyses with bootstrapping were used to examine the associations of emotion dysregulation with internalizing and externalizing symptoms in the entire sample as well as the groups. Fischer *Z*-Transformations of *r* were used to test whether there were significant differences in the strength of correlations between groups. Finally, hierarchical multiple regressions were used to examine whether emotion regulation significantly predicts internalizing and externalizing symptoms above and beyond diagnoses.

## Results

### Participant Groups

The four groups (TD, ADHD, ASD, and Anxiety) did not differ significantly on age, χ^2^ = 3.44, *p* = 0.329 (see Table 1 for *n* of each group). The groups differed significantly on gender, χ^2^ = 32.77, *p* < 0.001, *phi* = 0.14. There were equal numbers of males and females in the TD group. As expected, the anxiety group had slightly more females than males, while both ADHD and ASD groups had larger numbers of males than females.

### Groups Differences on Emotion Regulation, Internalizing, and Externalizing Symptoms

**Figure 1:**
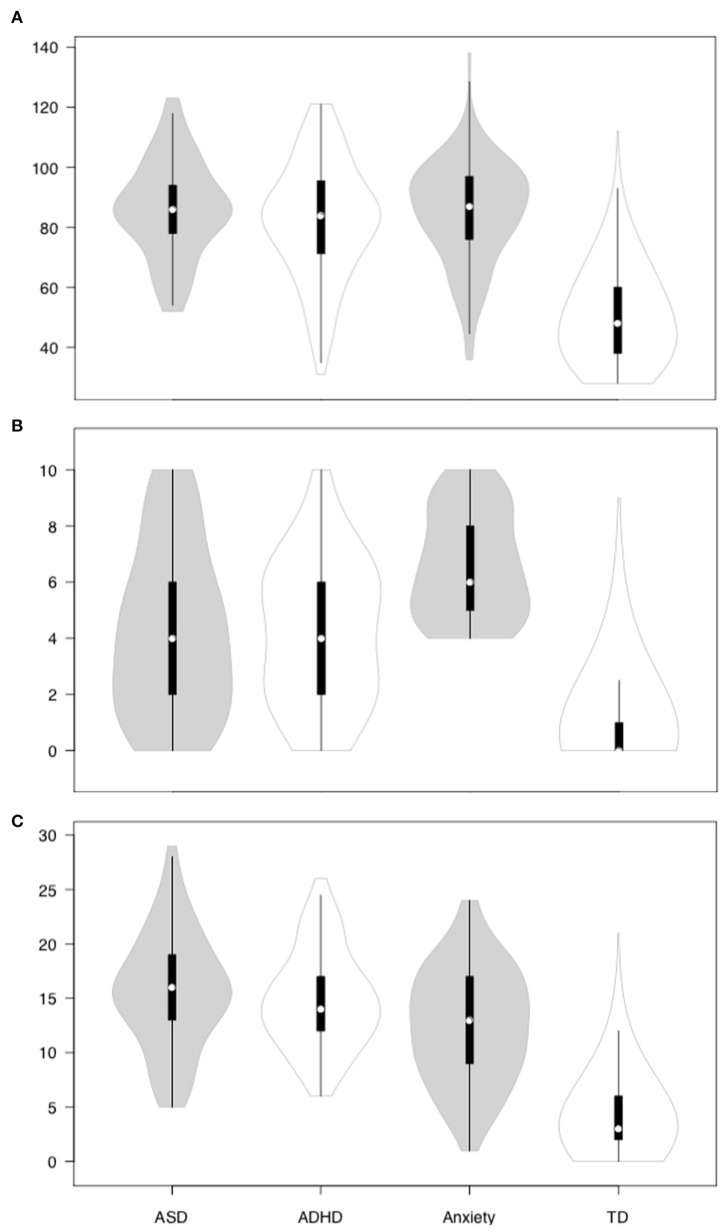
DERS, internalizing and externalizing symptoms across the clinical and non-clinical subgroups. **(A)** DERS; **(B)** internalizing symptoms; **(C)** externalizing symptoms.

The average scores on the DERS for the four subgroups were: TD (*M* = 50.06; *SD* = 15.07), Anxiety (*M* = 84.96, *SD* = 16.96), ADHD (*M* = 82.23, *SD* = 19.35), and ASD (*M* = 86.00, *SD* = 15.85). The distribution of DERS scores is presented in Figure 1A.

There was a significant group effect for the DERS scores, χ^2^ = 557, *p* < 0.001. Specifically, the TD subgroup had significantly lower DERS scores when compared to the subgroup with parent-reported anxiety diagnosis (hereinafter anxiety subgroup), *U* = 11,457, *z* = −15.10, *p* < 0.001, *r* = 0.39, ADHD, *U* = 16,651, *z* = −14.37, *p* < 0.001, *r* = 0.37, and ASD subgroups, *U* = 7,536, *z* = −14.27, *p* < 0.001, *r* = 0.37. The clinical subgroups did not differ significantly on DERS.

The four subgroups also differed significantly on internalizing, χ^2^ = 593, *p* < 0.001 (see Figure 1B), and externalizing symptoms, χ^2^ = 642, *p* < 0.001 (see Figure 1C). The anxiety subgroup (*M* = 6.60, *SD* = 1.91) reported significantly higher internalizing symptoms than the ADHD subgroup (*M* = 4.18, *SD* = 2.54), *U* = 3,276, *z* = −6.73, *p* < 0.001, *r* = 0.44, and the ASD subgroup (*M* = 4.20, *SD* = 2.96), *U* = 2,687, *z* = −6.07, *p* < 0.001, *r* = 0.42. The ADHD and ASD subgroups in turn reported significantly higher internalizing symptoms than the TD subgroup (*M* = 0.92, *SD* = 1.44), *U*_*ADHD*_ = 22,022, *z* = −14.40, *p* < 0.001, *r* = 0.37, and *U*_*ASD*_ = 19,957, *z* = −12.24, *p* < 0.001, *r* = 0.32. In contrast, the ADHD (*M* = 14.81, *SD* = 4.49) and ASD (*M* = 15.59, *SD* = 5.23) subgroups reported significantly higher externalizing symptoms than the anxiety subgroup (*M* = 12.50, *SD* = 5.20), *U*_*ADHD*_ = 5,052, *z* = −3.17, *p* = 0.002, *r* = 0.21, and *U*_*ASD*_ = 3,604, *z* = −3.89, *p* < 0.001, *r* = 0.27. The anxiety subgroup in turn reported significantly higher externalizing symptoms than the TD subgroup (*M* = 4.09, *SD* = 3.32), *U* = 13,959, *z* = −14.60, *p* < 0.001, *r* = 0.38. The ADHD and ASD subgroups did not differ significantly on internalizing and externalizing symptoms.

### Relationship Between Emotion Regulation and Internalizing and Externalizing Symptoms

Correlation analyses showed that emotion dysregulation was significantly and positively associated with internalizing and externalizing symptoms in the entire sample as well as the TD, Anxious, ADHD and ASD subgroups (see Table 2).

**Table 2:** Correlations with emotion regulation by group.

	**Internalizing**	**Externalizing**
	***r***	***r***
Whole sample	0.66^*^	0.75^*^
TD	0.36^*^	0.52^*^
Anxious	0.45^*^	0.61^*^
ADHD	0.53^*^	0.56^*^
ASD	0.63^*^	0.52^*^

* *p < 0.001*.

Although the direction of the relationship between emotion dysregulation and internalizing and externalizing symptoms was identical across all groups, Fischer *Z*-Transformations demonstrated that the emotion dysregulation-internalizing symptoms relationship was significantly stronger in ASD than TD (*z* = −3.35, *p* < 0.001) and anxiety subgroups (*z* = −1.81, *p* = 0.035). The strength of correlations for the ASD and ADHD subgroups were not significantly different. The strengths of the correlations between emotion dysregulation and externalizing symptoms did not differ significantly between the four subgroups.

The regression analyses indicated that emotion dysregulation significantly and positively predicted (statistically) internalizing and externalizing symptoms across the four subgroups (see Table 3).

**Table 3:** Regression results by subgroup: emotion regulation predicting internalizing and externalizing symptoms.

	**Internalizing**	**Externalizing**
	***F*, *p*, variance**	***F*, *p*, variance**
TD	206.25, *p* < 0.001, 13.0%	516.97, *p* < 0.001, 27.3%
Anxiety	27.42, *p* < 0.001, 19.8%	66.99, *p* < 0.001, 37.6%
ADHD	45.85, *p* < 0.001, 28.3%	53.89, *p* < 0.001, 31.6%
ASD	59.21, *p* < 0.001, 39.4%	33.75, *p* < 0.001, 27.1%

Two hierarchical multiple regression analyses showed that at step 1, diagnosis significantly predicted 29.2% of the variance in internalizing symptoms and 49.9% of the variance in externalizing symptoms for the whole sample of adolescents. At step 2, emotion regulation predicted additional 17.8% and 16.5% variances in internalizing and externalizing symptoms, respectively (see Table 4).

**Table 4:** Hierarchical multiple regressions of diagnosis and emotion regulation predicting internalizing and externalizing symptoms in the whole sample.

	***R*^**2**^**	***R*^**2**^ change**	***B***	**SEB**	**β**	***t***	***p***
**Internalizing**							
Model 1	0.292						
Diagnosis			−1.55	0.06	−0.54	−26.49	0.000
Model 2	0.470	0.178					
Diagnosis			−0.65	0.06	−0.23	−10.39	0.000
Emotion regulation			0.06	0.00	0.52	23.91	0.000
**Externalizing**							
Model 1	0.499						
Diagnosis			−4.61	0.11	−0.71	−41.21	0.000
Model 2	0.664	0.165					
Diagnosis			−2.65	0.11	−0.41	−23.26	0.000
Emotion regulation			0.134	0.01	0.50	28.86	0.000

## Discussion

Emotion regulation has been suggested as a key process that plays a pivotal role in developing and maintaining internalizing and externalizing symptoms (50, 51). Although several studies have demonstrated this link within specific categorically defined disorders, no study to date has attempted to characterize potential continuities and discontinuities in the nature of the association between emotion regulation with internalizing and externalizing symptoms across the normative-clinical continuum. Given the key implications of this work on informing treatment approaches, the current investigation aimed to determine if emotion dysregulation is positively related to and predicts internalizing and externalizing symptoms across a sample consisting of normative and clinical (neuropsychiatric and neurodevelopmental conditions) subgroups of adolescents.

As expected, the study's findings supported our first hypothesis that TD adolescents would have significantly better caregiver-reported emotion regulation than adolescents with caregiver-reported neuropsychiatric (anxiety) and neurodevelopmental (ASD and ADHD) conditions. This finding aligns with previous work showing that individuals with anxiety, ASD, or ADHD have poorer emotion regulation than those without neuropsychiatric or neurodevelopmental disorders (27, 52, 53). We also observed that adolescents from the clinical subgroups did not differ on emotion dysregulation.

The findings also supported our second hypothesis; emotion dysregulation significantly and positively correlated with and predicted internalizing and externalizing symptoms for each subgroup (TD, anxious, ADHD, and ASD). Emotion dysregulation accounted for a relatively large proportion of variance in both internalizing (18%) and externalizing (17%) symptoms across the four groups. It is interesting to note that although the anxiety subgroup reported the highest levels of internalizing symptoms compared with the other subgroups and emotion dysregulation did not differ across the clinical subgroups, emotion dysregulation was more strongly correlated with internalizing symptoms for the ASD and ADHD subgroups. This finding indicates emotion dysregulation plays a more prominent role in internalizing symptoms for adolescents with ASD and ADHD than typically developing adolescents and adolescents with anxiety. There is a plethora of evidence demonstrating the presence of executive dysfunction in individuals with ADHD and ASD. Children with ADHD and ASD share deficits in components of executive functioning, such as attention, working memory, fluency, preparatory processes, and concept formation (54). Importantly, studies have suggested that children with ADHD and ASD show poorer performance on executive functioning related tasks when compared to children with anxiety and depression (55). Given that individual differences in executive functioning predict the ability to regulate emotions (56), the stronger relationship between emotion dysregulation and internalizing symptoms observed in our adolescents with ASD and ADHD may be due to the additional executive functioning deficits experienced by these two groups. The strengths of relationships between emotion dysregulation and externalizing symptoms did not differ significantly between the four subgroups.

Crucially, we found that emotion dysregulation had a unique contribution to individual differences in the severity of internalizing and externalizing symptoms, above and beyond the diagnostic status. Emotion dysregulation accounted for a relatively large proportion of variance in both internalizing (18%) and externalizing (17%) symptoms across the four groups over and above diagnoses. We included the diagnoses of adolescents in the hierarchical multiple regression because we wanted to distinguish categorical classifications and dimensional measures of psychopathology. There were two possible results. The first possibility was that ER did not predict additional variances in internalizing and externalizing symptoms (dimensional measures) above and beyond the diagnoses (categorical classifications), which suggests symptoms can be wholly accounted for by the clinical diagnoses and ER was not an additional transdiagnostic factor of symptoms. The other possibility was that ER did predict additional variances in internalizing and externalizing symptoms above and beyond the diagnoses, which was what we have found in our study. Our findings indicate even though there is much overlap between symptoms of psychopathology and diagnoses, emotion dysregulation provided additional contribution to the symptoms. More specifically, our findings highlight the importance of assessing ER irrespective of the primary diagnosis, and that from the treatment perspective, if impaired, ER should be targeted regardless of the specific diagnostic classification.

Several limitations of the study are important to note. Although we explored the relationships between emotion dysregulation and internalizing and externalizing symptoms, this study's cross-sectional nature does not allow us to infer causal relationships. Due to the online survey design, it was not possible to independently verify the participant's diagnostic status *via* established and in-person diagnostic instruments (e.g., Autism Diagnostic Observation Schedule and Autism Diagnostic Interview-Revised). However, only participants who met the cut-off criteria on specific screening/quantitative severity instruments that show an optimal balance between sensitivity and specificity were included. It was also not possible to conduct IQ assessments of adolescents. Further, this study relied on caregiver-report measures of emotion dysregulation, internalizing and externalizing symptoms. The emotional symptoms subscale of SDQ was used to confirm children's diagnosis of anxiety. Although this subscale is not specific to anxiety, it has been shown to perform well to screen for anxiety in children (57). Due to the participant burden, it was not feasible to include more comprehensive assessments. Although unique in its transdiagnostic focus, this study nevertheless only included three clinical conditions. Future research will need to characterize further the relationships established in this study *via* longitudinal designs to determine the causality. In addition to longitudinal design, it will be important to further replicate and extend current findings through the use of in-person diagnostic and multimodal assessments of emotion regulation abilities and psychopathology. It would be relevant to examine factors that may impact the relationship between adolescent emotion regulation and psychopathology, such as other adolescent factors (IQ, language ability) and parent factors (quality of parent-child relationship, life stressors, parent emotion regulation, and psychopathology). Future research should also focus on subprocesses of ER to further advance our understanding of the association between ER and psychopathology.

The present findings have important research and translational implications. The neurobiology of emotion dysregulation may be similar across some or all of the conditions examined in this study. However, currently, there may not be enough evidence to conclude this. For instance, researchers have examined the neural correlates of cognitive reappraisal, a generally adaptive emotion regulation strategy in people with various conditions. Adults with ASD, anxiety or mood disorders showed less activation in the prefrontal cortex (PFC), specifically bilateral dorsolateral PFC in ASD and dorsomedial and ventrolateral PFC in anxiety and mood disorders (58, 59). In contrast, a recent study did not find any significant activation differences in the PFC between adults with ADHD and controls (60). Further work is needed to expand the current findings by investigating the continuities and discontinuities of the neurobiological underpinnings of emotion regulation across normative and clinical populations.

Given that the directions of the relationships between emotion dysregulation and symptoms are consistent across the TD and clinical subgroups, it is likely that interventions aiming to improve emotion regulation would reduce internalizing and externalizing symptoms of patients diagnosed with various clinical conditions. Several treatment trials support this suggestion. For example, Sakiris and Berle (61) conducted a systematic review and meta-analysis of Unified Protocol for Transdiagnostic Treatment of Emotional Disorders, an emotion regulation based intervention. They found large effective size reductions of various internalizing symptoms such as anxiety, depression, panic disorder, and obsessive-compulsive disorders in participants post-intervention using findings from 15 studies. They also showed that the use of adaptive emotion regulation strategies increased, and maladaptive emotion regulation strategies decreased, with moderate effect sizes. Given the findings reported here, it will be essential to evaluate the effectiveness of emotion regulation based interventions for improving emotion regulation and internalizing and externalizing symptoms across neurodevelopmental and neuropsychiatric conditions.

## Data Availability Statement

The raw data supporting the conclusions of this article will be made available by the authors, without undue reservation.

## Ethics Statement

The studies involving human participants were reviewed and approved by Stanford University IRB. The patients/participants provided their written informed consent to participate in this study.

## Author Contributions

MU and AH designed the study and collected the data. MU and RC had access to the data. RC conducted the analyses and drafted the initial manuscript. All authors critically reviewed and provided feedback on the initial version of the manuscript and approved the final version of the manuscript.

## Conflict of Interest

The authors declare that the research was conducted in the absence of any commercial or financial relationships that could be construed as a potential conflict of interest.
